# Effect of osteopathic manipulative treatment on length of stay in a population of preterm infants: a randomized controlled trial

**DOI:** 10.1186/1471-2431-13-65

**Published:** 2013-04-26

**Authors:** Francesco Cerritelli, Gianfranco Pizzolorusso, Francesco Ciardelli, Emiliano La Mola, Vincenzo Cozzolino, Cinzia Renzetti, Carmine D’Incecco, Paola Fusilli, Giuseppe Sabatino, Gina Barlafante

**Affiliations:** 1 EBOM – European Institute for Evidence Based Osteopathic Medicine, Chieti, Italy; 2 AIOT – Accademia Italiana Osteopatia Tradizionale, Pescara, Italy; 3 Neonatal Intensive Care Unit - “Spirito Santo” Civil Hospital, Pescara, Italy; 4 Neonatology and Neonatal Intensive Care Unit, University of Chieti

**Keywords:** Osteopathic manipulative treatment, Newborns, Preterm infants, Length of stay, Costs, Weight gain

## Abstract

**Background:**

The use of osteopathic manipulative treatment (OMT) in preterm infants has been documented and results from previous studies suggest the association between OMT and length of stay (LOS) reduction, as well as significant improvements in several clinical outcomes. The aim of the present study is to investigate the effect of OMT on LOS in premature infants.

**Methods:**

A randomized controlled trial was conducted on preterm newborns admitted to a single NICU between 2008-2009. N=110 subjects free of medical complications and with gestational age >28 and < 38 weeks were enrolled and randomized in two groups: study group (N=55) and control group (N=55). All subjects received routine pediatric care and OMT was performed to the study group for the entire period of hospitalization. Endpoints of the study included differences in LOS and daily weight gain.

**Results:**

Results showed a significant association between OMT and LOS reduction (mean difference between treated and control group: -5.906; 95% C.I. -7.944, -3.869; p<0.001). OMT was not associated to any change in daily weight gain.

**Conclusions:**

The present study suggests that OMT may have an important role in the management of preterm infants hospitalization.

**Trial registration:**

ClinicalTrials.gov, NCT01544257.

## Background

Preterm birth is defined as an early delivery made before the 37th week of gestational age [1-3]. The burden of preterm newborns has been steadily rising worldwide during the last 20 years [3]. In 2005 the global rate of preterm newborns was 9.6% (95% C.I. 9.1–10.1) per 12.6 million births per year [3]. This rate differs across regions, with the highest proportion of preterm births in Africa (11.9%; 11.1–12.6) and the lowest in Europe (6.2%; 5.8–6.7) [3].

Preterm birth leads to an increased lifetime morbidity and healthcare costs due to higher rates of neurological, respiratory, cardiovascular and psychological disorders [4-6].

With regards to economic costs, Russell et al. (2007) estimated the annual cost of preterm-birth on NICU to be 5.8 billion dollars (US), corresponding to 47% of the overall cost for all infants hospitalization and to 27% of all pediatric stays [7]. Data from another study carried out in the UK on preterm infants born at 30 weeks, documented mean costs per length of stay (LOS) for premature infant being higher than £10,000 (~$15,500) compared to £1300 (~$2080) for term infant [8,9].

LOS seems also to be strongly related to the infant’s physiological status [10], in terms of cardiorespiratory functions, full enteral feeding (bottle and/or nipple) and stooling. These preterms’ growth indicators are clinically relevant, in term of achievements that must be reached before they are safely discharged home [10]. The majority of these physiological milestones are achieved between 34 and 36 weeks of post-menstrual age, although these are secondary to the patient’s clinical condition [10]. The same authors also pointed out that feeding and oxygen goals are usually achieved last compared to all others. As a consequence, all of these factors have a significant influence on LOS. Therefore, taking into account the above aspects of the care of preterm infant, LOS may be considered an important outcome in relation to both the clinical status of the patient and the economic impact of services provided by NICUs.

As far as complementary and alternative medicines are concerned, few studies have been conducted in the preterm infant population. A review on massage therapy showed that the application of a 10-15 min massage led to an improvement of weight gain and to a LOS reduction [11]. However, Cochrane’s review suggested that these findings were inconsistent and highlighted many methodological concerns with previous studies [12].

Furthermore, from an osteopathic medicine perspective, there are no randomized control trials on premature infants. In 2011 Pizzolorusso et al. carried out an observational study of N=352 infants, suggesting that osteopathic manipulative treatment (OMT) may play an important role in reducing the risks for LOS longer than 28 days (adjusted OR=0.45;0.26-0.74) and the number of episodes of vomit, regurgitation, gastric residual and enema (adjusted OR=0.22;0.09-0.51) [13].

The aim of this study is to confirm and investigate, using gold standard methods, the effectiveness of OMT in LOS in a population of preterm infants.

## Methods

### Ethics statement

Written informed consent was obtained for all subjects during study enrollment and the study was approved by the institutional review board of Pescara’s hospital. This trial has been registered at http://clinicaltrials.gov/ (identifier NCT01544257).

### Objectives

A single blinded randomized control trial was carried out in the NICU of Santo Spirito Hospital in Pescara, Italy, in the period between August 2008 and December 2009. The primary objective of the study was to evaluate the effectiveness of OMT in reducing LOS in a sample of premature infants. The secondary outcomes of the study were the difference in daily weight gain and NICU cost analysis.

### Osteopathic care

Osteopathic medicine is a form of drug-free non-invasive manual medicine, designated as complementary and alternative medicine (CAM). It relies on manual contact for diagnosis and treatment. It respects the relationship between body, mind and spirit in health and disease; it lays emphasis on the structural and functional integrity of the body and the body's intrinsic tendency for self-healing. Osteopathic practitioners use a wide variety of therapeutic manual techniques to improve physiological function and restore homeostasis that has been altered by somatic (body framework) dysfunction (ICD-10-CM Diagnosis Code M99.00-09). Related components of the somatic system include: skeletal; arthrodial and myofascial structures; vascular; lymphatic and neural elements, which can become impaired or altered.

Osteopathic practitioners use their understanding of the relationship between structure and function to optimize the body’s self-regulation and self-healing capabilities. This holistic approach to patient care and healing is based on the concept that a human being is a dynamic functional unit, in which all parts are interrelated and possesses its own self-regulatory and self-healing mechanisms.

Two essential components of osteopathic health care are the structural evaluation of the patient for diagnosis and an array of manipulative techniques for treatment.

The aim of the structural examination is to locate the somatic disfunction. In newborns the structural exam is usually performed with the infant lying down on the table. Diagnostic criteria for somatic disfunction are focused on tissue texture abnormalities and tone. Areas of asymmetry and misalignment of bony landmarks are evaluated. The quality of motion, its balance and organization are noted.

In treating children during the very first days of life osteopaths use a wide variety of therapeutic manual techniques to increase range of motion, to improve physiological function and/or support homeostasis that has been altered by somatic dysfunction.

The term osteopathic manipulative treatment (OMT) currently encompasses more than twenty types of osteopath-performed manual treatments [14]. The OMT techniques of choice in treating preterm infants are myofascial release, balanced ligamentous/membranous tension, indirect fluidic and v-spread [13,15].

In the present study, 8 osteopathic practitioners were involved and randomly divided in two groups: 4 osteopaths performing the evaluation (group A), and 4 osteopaths performing the evaluation and the treatment (group B). Osteopaths from group A and B entered to the NICU in different hours of the schedule days, to provide blinding and to avoid possible confounding. None of the osteopathic practitioners were involved in the study design, data entry or statistical analysis. In addition all practitioners, except for the treating osteopath, were unaware of patients allocation. Osteopathic service was provided twice a week, on Tuesdays and Fridays.

### Study population

All newborns who met the following inclusion criteria were considered eligible for the trial: male and female preterm infants entering the NICU in the period between August 2008 and October 2009, subjects born in the Pescara’s hospital and are free from medical complications with written informed consent from parents or legal guardians. Out of N=559 newborns entering the NICU, N= 220 met the inclusion criteria. The exclusion criteria were applied at entry and during the entire newborn’s LOS. At entry the application of the supplementary exclusion the criteria (list of exclusion criteria) further reduced the number of preterm infants to N=110.

List of exclusion criteria:

gestational age <29, >37 weeks;

osteopathic treatment performed >14 days after birth;

newborn transferred to/from other hospital;

newborn from to HIV seropositive and/or drug addict mother;

newborn with genetic disorders, congenital abnormalities, cardiovascular abnormalities, neurological disorders, proven or suspected abdominal obstruction, pre- and/or post-surgery patients, pneumoperitoneum and/or atelectasis;

newborn with respiratory distress syndrome.

Using a permuted-block randomization procedure (1:1 ratio), patients were sequentially allocated to the experimental and control arms using R software (version 2.11.0) as computer random number generator [16].

**Figure 1:**
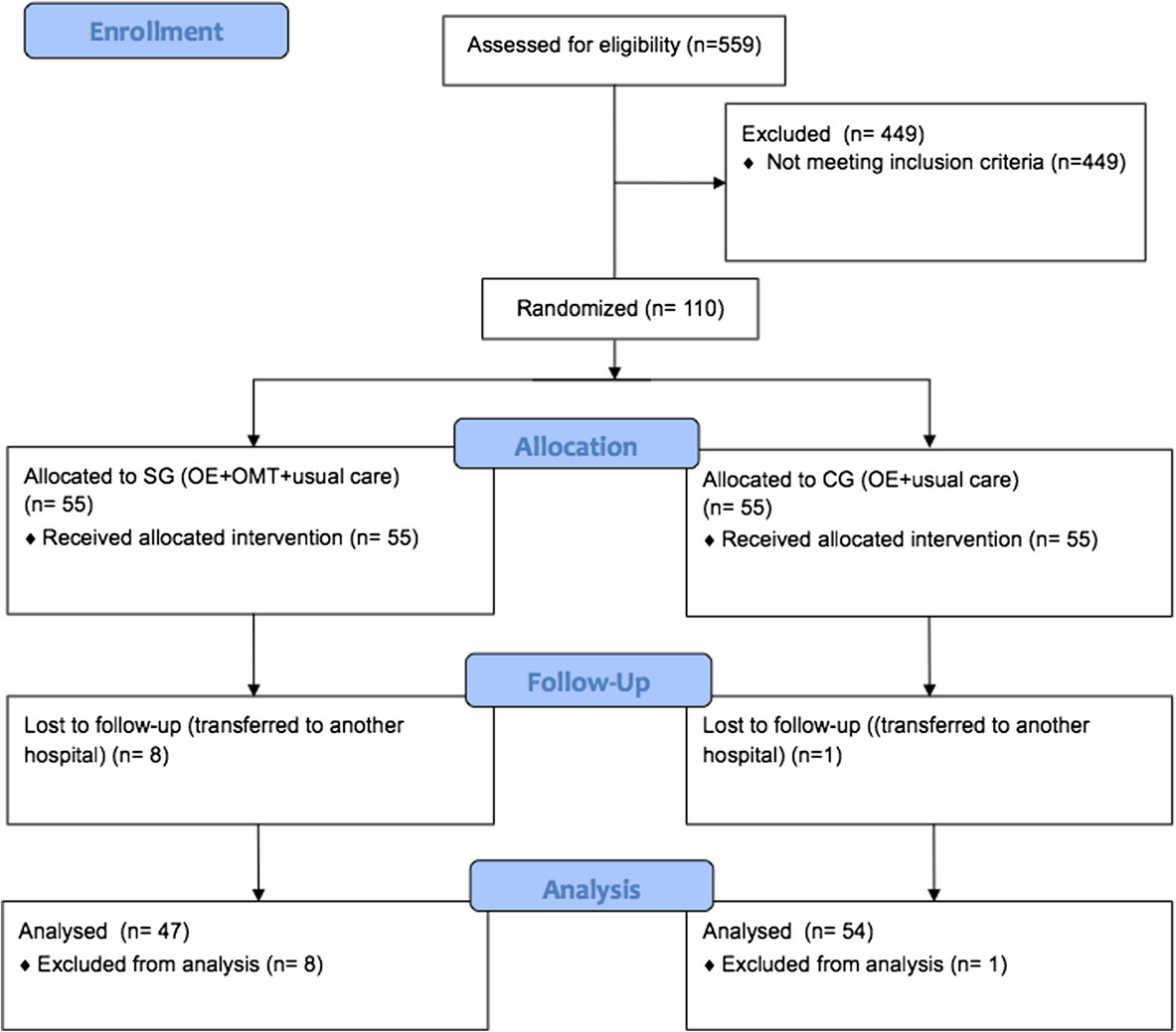
Flowchart for the selection of the study population.

N=55 preterm infants were randomly assigned to the experimental group, while N=55 infants to the control group (Figure 1). During the trial, 9 subjects dropped out transferred to another hospitals. At the end of the study N=47 preterms were allocated to the experimental group and N=54 to the control group.

Experimental group: patients under standard medical care plus OMT.

When osteopathic treatment was provided, patients from the experimental group received osteopathic evaluations and treatments from group B, plus osteopathic evaluations from group A. Group A was blinded to patients assignment and assessment. The osteopathic evaluation consisted of finding the somatic dysfunctions using a structural examination whilst the treatment was based on the application of indirect techniques as described above. The total time for osteopathic evaluation and treatment was 20 minutes.

Control group: patients under standard medical care plus osteopathic evaluations.

Patients from control group received only osteopathic evaluations from group A. The evaluation took approximately 10 minutes. Once completed, osteopaths from group A remained standing in front of incubators or beds for additional 10 minutes.

### NICU discharge criteria

Physiological conditions required for discharge included: maintenance of body heat at room temperature, coordinated sucking, swallowing, and breathing while feeding; sustained pattern of weight gain; and stability of cardiorespiratory function (no episodes of apnea/bradycardia for 2-5 days, free of supplemental oxygen support) [17].

### Allocation concealment and blinding

An information technology consultant was responsible for randomization prior to the arrival of the osteopaths to the NICU.

NICU staff was blinded to patients allocations, since all infants were at least touched by osteopaths from group A and B and osteopaths spent the same amount of time in front of incubators and/or beds. Moreover osteopaths were unaware of study design and outcomes.

### Data entering and data export

Data collection was performed using an ad hoc locally developed software called EBOM-GCCN.

EBOM-GCCN data set is an informatics tool that improves the efficiency and accuracy of data and developed to assist neonatologists, nurses and osteopaths in daily patient management.

The software consists of three sections:

Section 1: Intended for use by neonatologists and nurses for recording patients' general details and all clinical information;

Section 2: Intended for use by osteopaths from group A;

Section 3: Intended for use by osteopaths from group B.

Sections 1, 2 and 3 were blinded to all other investigator groups.

Nurses and medical records were collected daily by the NICU staff, from the time the infants were admitted to the NICU to the time of discharge. Each day the osteopathic service was provided, osteopaths from groups A and B recorded data in relation to the structural examination and OMT.

Data export was performed at the end of the study by the statistician from the coordinating center: European Institute for Evidence Based Osteopathic Medicine (EBOM).

### Statistical analyses

Sample size calculation used an effect size of 0.6 (mean groups difference of 6 days with a standard deviation of 10), a statistical power of 0.80 and an alfa level equal to 0.05. This produces a sample size of 45 per group. To prevent loss of power, the sample size was increased up to 55 subjects per group.

Statistical analyses are based on intention-to-treat analysis (ITT). Missing data is handled using last observation carried forward (LOCF) imputation technique. Arithmetic means and standard deviation are used for the general characteristics of the study population. Univariate statistical tests are used to compare the experimental group and control group at the baseline. A generalized linear model was also performed to study the independent effect of OMT on LOS and weight gain while taking into account possible confounders. The significance level is set at α=0.05.

The statistical program used for data analyses was R (version 2.11.0) [16].

### Cost analyses

A multivariate analysis was performed to study the average hospitalization costs among infants of study and control groups. Hospital discharge data are regularly collected by the Regional Office of the Ministry of Health (ROMH) of Abruzzo, where the NICU of the present study is located. In more detail, the Ministry of Health, besides its central offices, is divided into Regional Offices distributed throughout Italy. In relation to their specific competences, each ROMH carries out activities of control and offers services to the population.

Cost data used in the statistical model were extracted from ROMH 2009 administrative databases. The statistical study of costs and related economic implications were based on discharges of preterm infants with major complications and are explained as following.

The Istituto Superiore di Sanità (ISS, National Healthcare Institute) estimates a precise amount of reimbursement for each DRG (Diagnosis-Related Group). Thus, hospitals receive national and/or regional refunds according to patients DRG. In the case of this study, all infants included were dropped into the DRG category of “preterm with major complications”, which produces a reimbursement of 7.450,09€ adjusted per LOS per newborn [18].

In the statistical analyses, the reference category was given to the control group and the reimbursement value was divided by LOS (mean), producing an estimate cost per newborn per LOS. The latter was used to theoretically compute the exact costs for the study group.

As for the study group, the cost for each OMT session, equal to 20,00€, was conservatively obtained by the charges of insurance companies [19].

Ordinary Least Squares (OLS) regression was used to obtain the effects of OMT on hospitalization costs after adjusting for gender, gestational age, LOS and weight at birth.

All cost estimates were adjusted for inflation to 2012 EUR€ using the Medical Component of the Consumer Price Index [20].

## Results

101 subjects completed the study. According to ITT analysis, 110 preterm infants were analyzed at baseline. The two arms were balanced at baseline since no statistical differences were found after the univariate tests were applied (Table 1).

**Table 1:** General characteristics of the study population at the baselin

	**Study group**	**Control group**	**P value**
N*	55 (50.0)	55 (50.0)	
Gender*			
Male	26 (47.3)	28 (50.9)	0.89
Female	29 (52.7)	27 (49.1)	
Gestational Age	34 (2.3)	34 (2.5)	0.98
Weight (grams)			
At Birth	2088 (498.6)	2234 (730.9)	0.24
≤1500*	9 (16.4)	10 (18.2)	0.92
>1500*	46 (83.6)	45 (81.8)	
At Study Enrollment (gr)	1893 (496.7)	1926 (713.8)	0.59
Patient age (day of life)	3.2 (2.3)	3.6 (2.5)	0.82

Numbers in table are mean±s.d.; p value from *t* test.

*n(%);p value from chi-square test.

Variables considered as possible confounders that were included into the model are: gender, gestational age, weight at birth, milk volume at study enrollment and OMT.

During the trial, all subjects received banked milk and 11 preterms (5 study group and 6 control group, *X*^2^=0.04, p=0.84) were additionally fed with breast milk.

At the end of the study, 9 subjects dropped out due to hospital relocation (Figure 1). Reasons were: confirmation of genetic disorders (3 subjects from OMT group), bacterial infection complications (2 subjects from OMT group), cerebral hemorrhage (1 subject from the OMT group and 1 from the control group), convulsion (1 subject from OMT group) and cerebral hypoxia (1 subject from OMT group).

The mean LOS average was 26.1±16.4 for the study group whilst 31.3±20.2 for the control group (p<0.03).

A generalized linear model was performed to detect the real difference in mean between the two groups, taking into account confounders for the main outcomes.

**Figure 2:**
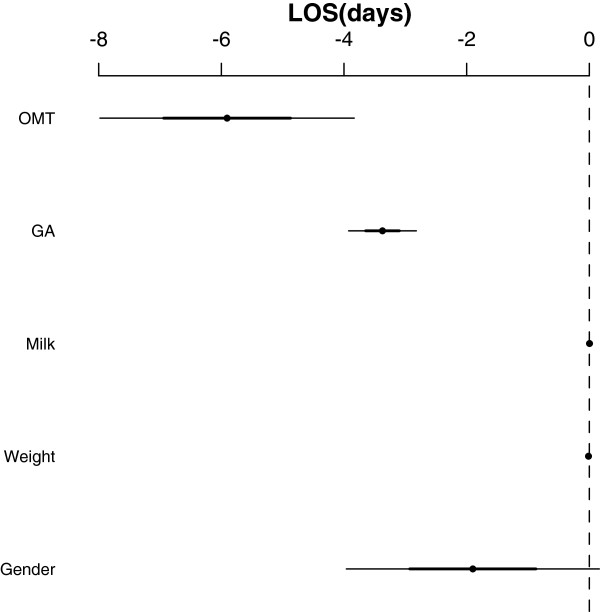
Multivariate linear regression for LOS.

Results for LOS are shown in Table 2 and Figure 2. All variables were found to be associated with a change in LOS, except for gender and milk volume at study enrollment.

**Table 2:** Results of multivariate linear regression

	**LOS (days)**			**Av. daily weight gain (gr)**		
	**β**	**95% C.I.**	**Pr(>|t|)**	**β**	**95% C.I.**	**Pr(>|t|)**
Male	-1.899	-3.930 , 0.127	0.07	0.708	-3.067 , 4.483	0.71
Gestational Age	-3.373	-3.916 , 2.830	<0.001	-0.338	-1.344 , 0.668	0.51
Birth Weight (gr)	-0.014	-0.016 , -0.009	<0.001	-0.018	-0.022 , -0.014	<0.001
Milk Volume at Study Enrollment (mL)	0.002	-0.004 , 0.009	0.44	0.059	0.045 , 0.072	<0.001
OMT	-5.906	-7.944 , -3.869	<0.001	3.707	-0.065 , 7.479	0.06

A negative association was found for gestational age (-3.373; 95% C.I. -3.916, - 2.830; p<0.001), birth weight (-0.014; -0.016, -0.009; p<0.001) and OMT (-5.906; -7.944, -3.869; p<0.001).

**Figure 3:**
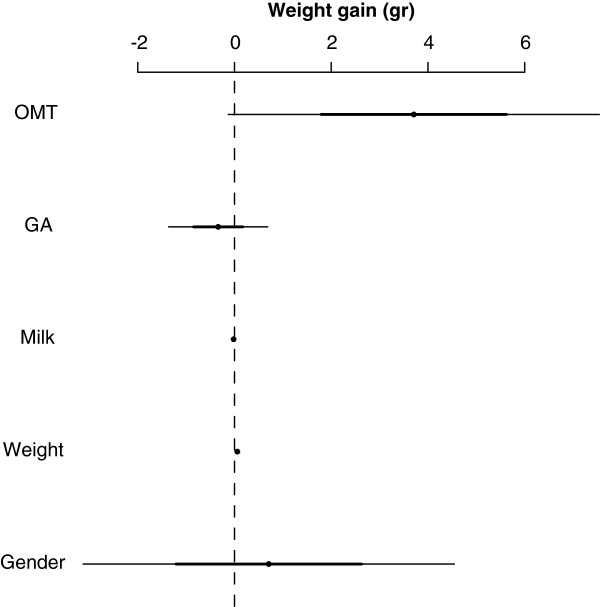
Multivariate linear regression for weight gain.

Results for weight gain are shown in Table 2 and Figure 3. Only birth weight (-0.018; -0.022, -0.014; p<0.001) and milk volume at admission (0.059; 0.045, 0.072; p<0.001) were found to be associated with changes in weight gain, while gender, gestational age and OMT were not statistically significant associated.

No adverse and side effects were shown in both groups during the study period.

### Cost analysis

The total expected NICU costs for preterm infant of study and control groups are shown in Table 3.

**Table 3:** Results of ordinary least square regression for cost estimates

	**Costs (2012€)**		
	**Estimate**	**95% C.I.**	**p>χ2**
Male	576.14	-173.65 , 1325.93	0.13
Gestational Age	-120.76	-371.79 , 130.27	0.34
Birth Weight (gr)	0.49	-0.43 , 1.43	0.28
LOS	78.96	38.17 , 119.75	<0.001
OMT	-2,724.91	-3,491.73 , -1,958.09	<0.001

Setting a cost of 20,00€ for each osteopathic treatment, the total expected NICU costs for infant from the OMT group and control group were 4,491.70€ and 7,450.09€ respectively, with a net direct saving of 2,958.39€ (95% C.I. 1,983.77, 3,933.00; p<0.001) for infant from the OMT group.

The adjusted OLS analysis (Table 3) shows that NICU costs are negatively associated with OMT (-2,724.91; -3,491.73, -1,958.09; p<0.001), thus producing a net savings, and positively associated with LOS (78.96; 38.17, 119.75;p<0.001), thus producing an increase in costs. All other variables used in the model did not produce a significant association with the NICU costs.

These estimates are based on cost per preterm per LOS. However, taking into account the entire OMT group and a study period of 15 months, the net saving, using OLS estimate values, is €139,044.30€ (93,237.19, 184,851.00).

## Discussion

The results from the present study highlight the potential benefit of OMT in newborns in our population. LOS reduction in the OMT group was highly significant compared to the standard care group (26.1 vs 31.3). This indicates that the OMT can shorten the period of hospitalization of almost 6 days, opening up several considerations from a health care prospective.

LOS has been widely used as an outcome measure in research contexts. Moreover, it has been considered a valid proxy measure to evaluate the clinical status in pre-term infants [10]. If the correlation between LOS and infant physiological status is considered to be strong [10], then these results would indicate that OMT in pre-term infants leads to significant benefits such as cost reductions, enhanced quality of health care service delivered and improved health status of pre-term infants.

Few studies are available in the medical literature in relation to the pediatric population and no structured OMT clinical trials have been carried out in preterm infants during their stay in NICUs.

Some data are available from studies on a wider population of infants and children, documenting the positive effects of osteopathic care in gastrointestinal function [21], obstructive apnea [22] and postural asymmetry [23].

There has been only one recent prospective study carried out in Italy [13], where the authors looked at the effect of OMT on LOS and gastrointestinal functions. Pizzolorusso et al showed that preterm infants treated with OMT group significantly reduced the hospital LOS (adjusted OR=0.22;0.09-0.51). Moreover, they showed that infants in the intervention group had a significance reduction in the risk of excessive gastrointestinal symptoms, such as vomit, regurgitation, gastric residual and enema administration (adjusted OR=0.45;0.26-0.74). These findings lead to consider a possible and potential role of OMT in increasing the global health condition of newborns. However, the study enrolled and analyzed all newborns accessing at the NICU without discriminating between pre-term and term neonates.

The mechanisms of action of OMT are not fully understood. Hypotheses based on clinical data are as follows. The first hypothesis takes into account the anti-inflammatory action of OMT. Narendran et al (2010) showed that prematures have a higher level of cortisol, albumin, IL-8 and IL-1β [24], suggesting an increased level of systemic inflammation. A study carried out in 2007 demonstrated that OMT can reduce the inflammatory process acting mainly on anti-inflammatory factors [25]. Moreover, Degenhardt et al suggested that OMT could have a role in increasing the opioid reaction [26]. Therefore OMT may potentially modulate and reduce the inflammatory status of infants through the anti-inflammatory mechanism. However, this hypothesis has some intrinsic limitations in terms of the sample used, i.e. no infants were osteopathically treated, and in translating vitro findings into vivo mechanisms.

Another possible hypothesis involves the function of the autonomic nervous system (ANS). Longin et al [27] demonstrated that gestational age of newborn infants is correlated with a change in the heart rate variability (HRV). Furthermore prematures have a different level of HRV ratio leading to considerably different reactions of the ANS. From the osteopathic perspective, changes in the HRV were recorded after the application of myofascial release techniques [28], as demonstrated by Henley et al. in 2008. For this reason, the application of OMT could bring balance to the sympathetic and para-sympathetic inputs, creating an improvement of newborns clinical condition. As for the previous hypotheses, the different sample does not allow to predict a similar reaction in preterm infants.

According to costs computation, the application of OMT can theoretically save almost 3,000€ per preterm per LOS, putting the NICU in a more favorable economic position. The latter is due to the ISS reimbursement procedures which establish a payment method based on DRG mean cost estimates [18] rather than on the duration of a single hospital admission. The net saving could be considered a “moneybox” that NICU managers can use to improve efficiency in procedures, implementing diagnostic tools or optimizing turnovers. The cost analysis is highly dependent upon the relationship of the cost-reimbursement of the OMT to the cost-reimbursement of the DRG. Considering the cost for OMT session equal to the cost of a standard physical care in a public domain (25€) [18], the net saving is approximately 2,700€ per LOS per newborn, confirming the osteopathic treatment a valuable intervention.

As a matter of fact, in a decentralized national health care system, osteopathy in NICUs can produce more benefits to the single regional and local health care policymakers rather than to ISS payment system, following the national criteria to propel more effective health care methods.

Some limitations of the present study need to be outlined, in terms of small sample size and all patients enrolled are referred to one single NICU and may be not representative of the preterm infants population. Moreover maternal socioeconomic status was not collected and it might be another potential confounder. Furthermore, during the study period, hospital relocation was predominately from the study group. This could be related to potential adverse effects of OMT, the type of the population, the relative small sample size and the fragile newborns’ conditions can casually predispose to this difference. Additional studies with lager sample size are needed to confirm these results.

As far as the economic evaluation is concerned, the cost estimation was based on a theoretical approach based on mathematical computation of hospital net savings. The major limitation of this approach is due to the national and regional reimbursement system which based its activity on diagnosis category (DRG) with a theoretical LOS threshold rather than on the real length of stay [18]. This produces theoretical savings that might not be representative of the real hospital scenario.

## Conclusions

The treatment of preterm infants with complementary and alternative medicine could be considered a potential and promising intervention to be included into the routine health care system. Results from the present study show the effectiveness of the OMT in reducing the length of stay, costs but not weight gain in a sample of pre-term newborns. Despite the lack of prospective research into the effects of osteopathy on preterm infants, our study shows strong evidence that OMT has a role in the care of newborns. Further studies based on multi-centric design are required to confirm our results.

## Abbreviations

OMT: osteopathic manipulative treatment; GA: gestational age; LOS: length of stay; NICU: neonatal intensive care unit; CAM: complementary and alternative medicine; HRV: heart rate variability

## Competing interests

All authors declare no conflict of interests to this paper. No financial assistance was received to any of the authors to conduct this study.

## Authors’ contributions

CeF, PG, BG planned the study, CF wrote the first draft and performed statistical analyses, CiF and LME wrote the protocol, CV, RC, DIC, FP, SG reviewed the manuscript. All authors have read and approved the submission of the manuscript.

## Pre-publication history

The pre-publication history for this paper can be accessed here:

http://www.biomedcentral.com/1471-2431/13/65/prepub
